# Notes and News

**Published:** 1898-07-30

**Authors:** 


### NOTES AND NEWS.

(Collected and Communicated.)

Professor W. H. Corfield has consented to become honorary sanitary adviser to the University College Hospital.

An announcement has been made that the Italian Government is about to construct sanatoria throughout the country for the purpose of treating tuberculous patients, and as a means of segregating them so as to minimise the spread of the disease among the poorer classes

Sir Samuel Wilks, President of the Royal College of Physicians, opened a cottage hospital at Emsworth last week. The hospital, built as a Jubilee memorial, is beautifully situated, and Sir Samuel Wilks, after a visit of inspection, expressed himself much pleased with the perfection of the arrangements.

The statements which have been so freely made concerning the mutilation of the American soldiers killed in action by the Spaniards have been very practically refuted by an examination of the Spanish soldiers wounded by the American bullets, in whom just the same sort of injuries have been discovered. Both the Mauser rifle, used by the Spanish troops, and the Krag-Jorgensen, used by the Americans, are small bore rifles, having a high initial velocity, and the wounds produced by each are of the same now well-recognised character.

We should very much like to know what foundation there is for the statement which appears in some of the American medical papers to the effect that the brass casings in which some of the Spanish bullets are found to be enclosed "can hardly fail to cause blood-poisoning in a person wounded by them, however slight the injury." It appears to us that this is a completely exaggerated view to take of the fact that copper and brass, when embedded in the tissues, do sometimes give rise to a certain degree of copper poisoning. In many cases copper coins have remained exposed even to intestinal juices for a long time without doing harm, and a not inconsiderable amount of slightly-plated copper wire has been used for sutures without evil consequences resulting.

Dr. Kingsbury is again in luck's way. The defendants in the recent trial have got to pay the costs.

H.R.H. the Prince of Wales continues to make satisfactory progress, and it has been decided that he will go to Cowes for the regatta week. It is understood that the position of the fragments of the broken patella has much improved.

In receiving a deputation which waited on him recently, urging him to take stringent action with regard to the use of certain kinds of phosphorus in match factories, and, at the same time, expressing a strong feeling that more women inspectors were needed, the Home Secretary announced his intention of appointing another woman inspector and a medical inspector, the sex of the latter not being specified.

The Duchess of Connaught opened a new hospital at Aldershot for soldiers' wives and children on Tuesday. The hospital is situated near the Cambridge Hospital in Stanhope Lines. It contains 53 beds—28 in the maternity block, and 25 in the general block. Of the 11 wards some are named after the Queen, the Duke of Connaught, and Lord Lansdowne. The building has cost £12,600.

A memorandum in regard to the formation of an association for the prevention of consumption and other forms of tuberculosis has been issued over the signatures of Samuel Wilks, President of the Royal College of Physicians, London; William MacCormac, President of the Royal College of Surgeons; W. H. Broadbent, M.D., F.R.S., Chairman of the Provisional Committee; and Malcolm Morris, hon. treasurer.

A curious complication has occurred in regard to the treatment of milk in creameries. Farmers can employ women on their farms, even on Sundays, to attend to milk, as a work of necessity; but creameries are factories, and Sunday labour in factories is not allowed. Mr. P. O'Brien asked the Home Secretary the other day whether he would take steps to legalise the employment of dairymaids in these factories on Sundays, and in the meantime would provide that the existing law should not be enforced against those engaged on Sundays in such work. Sir M. W. Ridley, in reply, said he thought there should be some such modification as was suggested in the Factory Acts.

A Bill has been introduced into the House of Commons which seeks to regulate the use of foreign university degrees in the United Kingdom. The Bill enacts that "any person who attaches to or who is responsible for the attachment to his name of the degree of bachelor, master, or doctor of any faculty in which degrees are granted, shall, unless such degree has been received from one of the universities of the United Kingdom of Great Britain and Ireland, or from any authority in the said United Kingdom which confers degrees by virtue of a Royal Charter, or by the sanction of the Crown or of Parliament, place and clearly indicate after such degree the source from which it has been received." The term "degree" is to mean and include any letters or abbreviations which are commonly used to signify the degree of bachelor, master, or doctor of any faculty in which degrees are granted. The Bill bears the names, among others, of Dr. Farquharson and Sir William Priestley.

